# Amyloid β Peptide Induces Apoptosis Through P2X7 Cell Death Receptor in Retinal Cells: Modulation by Marine Omega-3 Fatty Acid DHA and EPA

**DOI:** 10.1007/s12010-015-1878-6

**Published:** 2015-10-14

**Authors:** Anaïs Wakx, Mélody Dutot, France Massicot, Frédéric Mascarelli, G. Astrid Limb, Patrice Rat

**Affiliations:** UMR CNRS 8638—Chimie-Toxicologie Analytique et Cellulaire, Sorbonne Paris Cité, Faculté de Pharmacie, Université Paris Descartes, 4 avenue de l’Observatoire, 75006 Paris, France; Laboratoire Yslab, 2 rue Félix Le Dantec, 29000 Quimper, France; INSERM U 872—Physiopathologie des maladies oculaires: Innovations thérapeutiques, Centre de Recherches des Cordeliers, 15 Rue de l’Ecole de Médecine, 75006 Paris, France; Division of Ocular Biology and Therapeutics, UCL Institute of Ophthalmology and Moorfields Eye Hospital, 11 Bath Street, London, EC1V 9EL UK; Inserm U598, Physiopathologie des maladies oculaires, Innovations thérapeutiques, Centre de Recherches Biomédicales des Cordeliers, 75270 Paris Cedex 06, France

**Keywords:** Age-related macular degeneration, P2X7 receptor, Amyloid-β peptide, Retinal cells, Apoptosis, DHA, EPA, Omega-3 fatty acid

## Abstract

Retinal Müller glial cells have already been implicated in age-related macular degeneration (AMD). AMD is characterized by accumulation of toxic amyloid-β peptide (Aβ); the question we raise is as follows: is P2X7 receptor, known to play an important role in several degenerative diseases, involved in Aβ toxicity on Müller cells? Retinal Müller glial cells were incubated with Aβ for 48 h. Cell viability was assessed using the alamarBlue assay and cytotoxicity using the lactate dehydrogenase (LDH) release assay. P2X7 receptor expression was highlighted by immunolabeling observed on confocal microscopy and its activation was evaluated by YO-PRO-1 assay. Hoechst 33342 was used to evaluate chromatin condensation, and caspases 8 and 3 activation was assessed using AMC assays. Lipid formulation rich in eicosapentaenoic acid (EPA) and docosahexaenoic acid (DHA) used in Age-Related Eye Disease Study 2 was incubated on cells for 15 min prior to Aβ incubation. For the first time, we showed that Aβ induced caspase-independent apoptosis through P2X7 receptor activation on our retinal model. DHA and EPA are polyunsaturated fatty acids recommended in food supplement to prevent AMD. We therefore modulated Aβ cytotoxicity using a lipid formulation rich in DHA and EPA to have a better understanding of the results observed in clinical studies. We showed that fish oil rich in EPA and DHA, in combination with a potent P2X7 receptor antagonist, represents an efficient modulator of Aβ toxicity and that P2X7 could be an interesting therapeutic target to prevent AMD.

**Graphical Abstract:**
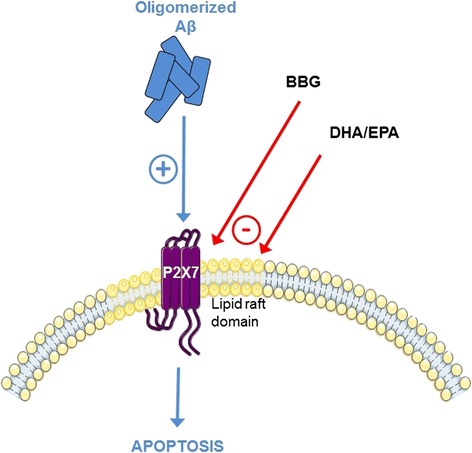
ᅟ

ᅟ

## Introduction

Age-related macular degeneration (AMD) is a progressive degeneration of the macula, the portion of the retina used for central vision. It is the leading cause of the irreversible loss of vision in those aged over 50 years in the Western industrialized world [1]. The United Nations estimates the number of people with AMD at 20–25 million worldwide [2]. As AMD progresses, it can develop into two distinct forms of late or advanced AMD: “dry” AMD (geographic atrophy, 90 %) and “wet” AMD (neovascular AMD, 10 %). Early stage of AMD is characterized by the formation of drusen that are deposits of extracellular material located underneath the retinal pigmented epithelium (RPE). Drusen provokes an inflammatory response and is associated with RPE atrophy. Photoreceptors overlying drusen die by apoptosis, whereas retinal Müller glial cells are activated. Under physiological conditions, Müller cells are responsible for maintaining its homeostasis, support neuronal activity, and participate in the induction, maintenance, and proper functioning of the blood–retinal barrier [3–5]. Alterations of Müller cells under pathological conditions can contribute to retinal degeneration [6–8]. Especially, Müller cell dysfunction leads to photoreceptor apoptosis and blood–retinal barrier breakdown [9, 10]. There is no curative treatment against atrophic AMD, which affects 90 % of AMD patients. Indeed, consumption of micronutrients, such as zinc, β-carotene, or vitamins, has been shown to prevent AMD progression. A study reviewing the role of dietary omega-3 long chain polyunsaturated fatty acid (PUFA) in the prevention of AMD reported a 38 % reduced rate of progression to late AMD [11]. docosahexaenoic acid (DHA, C22:6 ω-3) and its precursor eicosapentaenoic acid (EPA, C20:5 ω-3) are the major structural long chain PUFAs of the membrane of photoreceptors [12]. DHA is essential for the biogenesis and the function of photoreceptors [13]. Moreover, EPA and DHA have antioxidant, anti-inflammatory, antiapoptotic, and antiangiogenic roles in the retina [14, 15]. PUFA content in the retina decreases with aging and it potentially induces a dysfunction of retinal cells. Participants who reported the highest levels of EPA consumption had a reduced likelihood of AMD progression [16]. Amyloid-β (Aβ) peptide is a key constituent of drusen [17–19]. It has been suggested that drusen could correspond to the transposition of senile plaques in Alzheimer’s disease (AD). In the retina of mice models of AD, an age-dependent Aβ accumulation has been detected, possibly resulting in neurodegeneration [20]. It has been found that oligomerized Aβ is more toxic than is nonoligomerized Aβ in retinal cell cultures [21, 22]. Retinal toxicity seems to be associated with oxidative stress and pro-inflammatory response, but underlying mechanisms remain not clearly defined [23, 24]. The purinergic receptor P2X7 is an ATP-gated cationic channel expressed by virtually all types of cells [25, 26]. P2X7 is involved in oxidative stress, cell death, and inflammatory processes, all of which have been linked to AMD [27, 28]. A recent genetic study has demonstrated that a haplotype containing a rare genetic variant of P2X7 receptor is associated with increased susceptibility to AMD [29].Moreover, Notomi et al. recently proposed Brilliant Blue G (BBG), a selective P2X7 receptor antagonist, as a neuroprotective agent in retinal diseases [30]. The first aim of our study was to describe the P2X7-dependent cell death pathway induced by Aβ on Müller cells. Our second aim was to modulate Aβ cytotoxicity using a lipid formulation rich in DHA and EPA, chosen for its ability to modulate toxic ocular stresses [31, 32].

## Methods

### Reagents

Reagents for cell culture were provided by Eurobio (Les Ulis, France), flasks and microplates from Corning (Schiphol-Rijk, The Netherlands) and chamber slides from Nunc Thermo Fisher Scientific (Rochester, NY, USA). Lipid formulation rich in DHA and EPA was provided by Yslab (Quimper, France). BBG, a specific P2X7 receptor inhibitor [33], was purchased from Bio-Rad (Richmond, CA, USA). Hoechst 33342, YO-PRO-1, TO-PRO-3, and secondary antibodies were purchased from Invitrogen (PoortGebouw, The Netherlands). Aβ (Bachem, Weil am Rhein, Germany) was oligomerized as previously described [34]. Primary antibodies and IgG isotype control were provided from Santa Cruz Biotechnology (Heidelberg, Germany). All other chemicals, dyes, and kits were provided from Sigma-Aldrich (Saint Quentin Fallavier, France).

### Cell Culture

The experiments were performed using the immortalized human Müller cell line MIO-M1 [35]. The MIO-M1 cell line was tested at IDEXX BioResearch (Columbia, MO, USA). The cell line was confirmed to be human and no evidence of cross-species contamination was found. The STR testing results reported for the cell line are as follows: amelogenin (X, Y), CSF1PO (13, 14), D13S317 (13), D16S539 (11, 12), D5S818 (12, 13), D7S820 (7, 9), TH01 (6, 9.3), TPOX (6, 9), and vWA (15, 19).

MIO-M1 were cultured using Dulbecco’s Modified Eagle’s medium, supplemented with 10 % fetal bovine serum (FBS), 2 mM L glutamine, 50 IU/mL penicillin, and 50 IU/mL streptomycin, at 37 °C in a humidified atmosphere of 5 % CO_2_, as previously described [36]. Confluent cultures in flasks were removed by trypsin incubation, and then the cells were seeded into 96-well (20,000 cells per well) or 24-well (38,000 cells per well) culture microplates and kept at 37 °C for 24 h.

### Incubation Protocols

Whenever the cells reached confluency, culture medium was removed and the cells were incubated with oligomerized Aβ 1–42 at 25 μM in 2.5 % FBS medium for 48 h at 37 °C. Excessive Aβ was used to model AMD pathology. Twenty-five micromolars was the highest Aβ concentration that did not induce any loss of cell viability in our model (data not shown).

Neat fish oil containing EPA and DHA (see composition in Table 1) was incubated for 15 min (100 μL/well) followed by 24 h in culture medium prior to Aβ incubation [31, 32, 37]. BBG (25 μM, according to Kawahara [38]) was incubated with cells for 15 min prior to Aβ incubation. BBG is a potent inhibitor of P2X7 receptor since concentrations as low as 25 μM are enough to inhibit receptor activation.

**Table 1:** EPA and DHA (%) and tocopherol (mg/g) composition of tested oil

	Fish YS-2636
C20:5 ω3 EPA	36
C22:6 ω3 DHA	26
Mixed tocopherol	3.6

### Cell Viability (Necrosis Assessment): AlamarBlue Assay

The alamarBlue^®^ assay uses resazurin, a blue fluorogen probe, which is reduced to a red fluorescent compound (resorufin) by intracellular redox enzymes [39]. A solution of resazurin at 0.1 mg/mL was prepared in phosphate-buffered saline (PBS) then diluted to the eleventh well in culture medium supplemented with 2.5 % FBS. The cells were exposed to resazurin solution for 6 h at 37 °C, then the fluorescence signal was read (λexc = 535 nm, λem = 600 nm, Safire; Tecan^®^, Zurich, Switzerland).

### Cytotoxicity: LDH Release Assay

The lactate dehydrogenase (LDH) assay measures membrane integrity as a function of the amount of cytoplasmic LDH released into the medium [40]. Briefly, cell supernatants were incubated with the LDH mixture containing NAD as LDH substrate and a tetrazolium dye (the mixture was prepared according to the manufacturer’s instructions for Sigma kit TOX7) for 30 min. Absorbance was detected at 490 nm (Safire; Tecan^®^, Zurich, Switzerland).

### P2X7 Expression by Immunofluorescence Using Confocal Microscopy

After seeding in chamber slides for 24 h, the cells were fixed with 2 % paraformaldehyde and 2 mM calcium for 15 min at room temperature. The cells were then permeabilized with 0.2 % Triton X-100 for 5 min. First, the cells were incubated with primary antibody (rabbit anti-P2X7 at 5 μg/mL) or rabbit IgG (isotype control) in PBS with 1 % bovine serum albumin (BSA) for 1 h, and second, the cells were incubated with secondary antibody (Alexa Fluor 488 anti-rabbit IgG) in PBS with 1 % BSA for 1 h away from light. Third, nuclei were stained with TO-PRO-3 at 2 μg/μL for 10 min. Slides were then observed under a Leica TCS SP2 confocal microscope (Leica Microsystems) equipped with a 40× oil immersion objective. Staining specificity was carefully checked by omitting the primary antibody.

Confocal imaging was performed at IFR71-IMTCE Cellular and Molecular Imaging platform (Faculté de Pharmacie, Université Paris Descartes, Paris, France).

### P2X7 Activation: YO-PRO-1 Test

YO-PRO-1, a fluorogenic probe, enters cells through P2X7 receptor activation-induced pores and emits fluorescence when it binds DNA [41]. A 2-μM YO-PRO-1 solution in PBS was distributed in wells, and the microplate was placed at room temperature away from light for 10 min [42–46]. The fluorescence signal was then scanned (λexc = 491 nm, λem = 509 nm, Safire; Tecan^®^, Zurich, Switzerland).

### Chromatin Condensation: Hoechst 33342 Assay

Hoechst 33342 dye is used to detect chromatin condensation in cells simultaneously with propidium iodide [44]. Hoechst 33342 enters living and apoptotic cells whereas propidium iodide enters necrotic cells faster than Hoechst 33342. A solution of Hoechst 33342 at 10 mg/mL and propidium iodide at 0.5 mg/mL was prepared in PBS. Cells were exposed for 30 min at 37 °C, then the fluorescence was read (λexc = 365 nm, λem = 450 nm, Safire; Tecan^®^, Zurich, Switzerland).

### Caspases 3 and 8 Activation: AMC Assays

The caspases 3 and 8 fluorometric assays were realized following the procedure for fluorometric assay of caspases 3 and 8 activity in adherent cell lines of the CASP3F and CASP8F Sigma kits. Briefly, the cells were treated with lysis buffer and incubated on ice for 20 min. Then, DEVD-AMC for caspase 3 detection or IETD-AMC for caspase 8 detection was added in each well. The samples were incubated in the dark at room temperature for 30 min. Afterward, the fluorescence signal was read (λexc = 360 nm, λem = 460 nm, Safire; Tecan^®^, Zurich, Switzerland).

### Results Exploitation and Statistical Analysis

All data for microtitration were obtained in fluorescence or absorbance units and expressed as a percentage of the negative control (culture medium). Each point was tested in three different wells, and experiments were reproduced in triplicate. Data are expressed as means ± standard deviation. The mean values for each test were analyzed by one-way ANOVA followed by the Dunnett test (SigmaStat 2.0; Chicago, Illinois, USA), and the level of significance was fixed at 0.05.

## Results

### Cell Viability in Aβ-Incubated MIO-M1 Cells

**Fig. 1:**
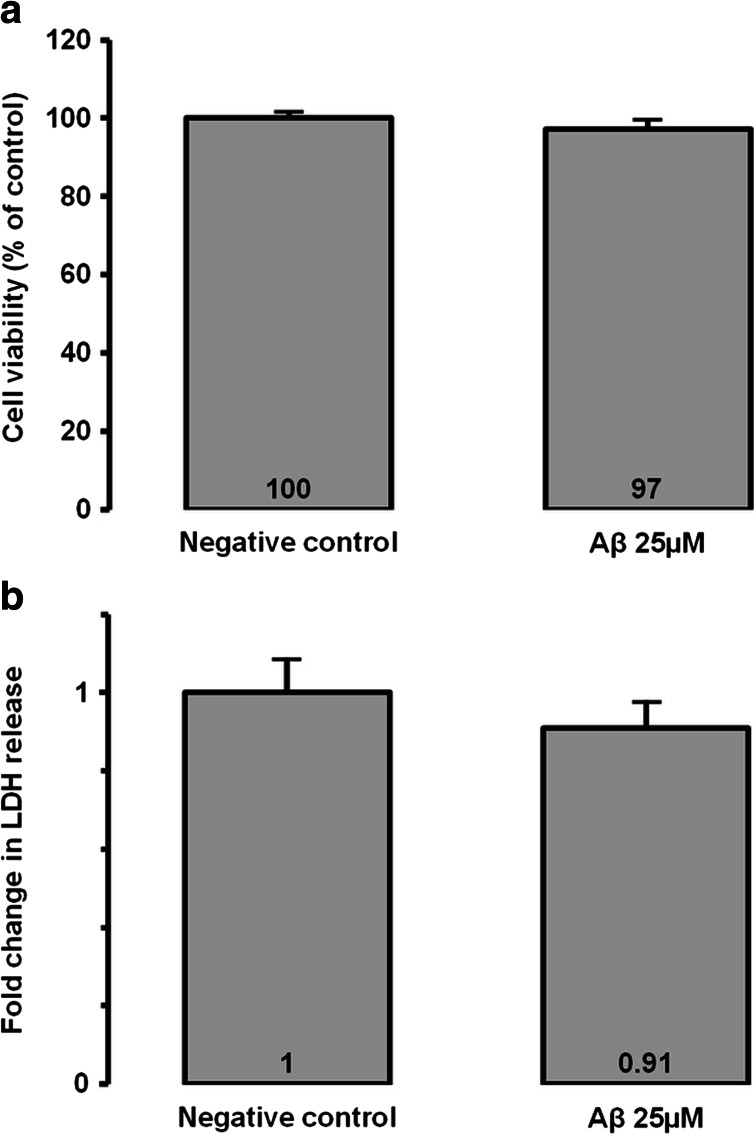
Cell viability and cytotoxicity assessment after incubation of 25 μM Aβ for 48 h. **a** Cell viability was assessed using global redox potential. **b** Necrosis was assessed using extracellular LDH dosage after Aβ incubation for 48 h in MIO-M1 retinal Müller cells

First, we investigated cell viability in Aβ-incubated MIO-M1 cells. Aβ at 25 μM did not induce any significant decrease in cell viability according to the alamarBlue assay (Fig. 1a). The LDH activity assay showed no significant change in the plasma membrane integrity which was detected in Aβ-incubated MIO-M1 in comparison to negative control (Fig. 1b).

### P2X7 Activation, Chromatin Condensation and Caspase Activity

**Fig. 2:**
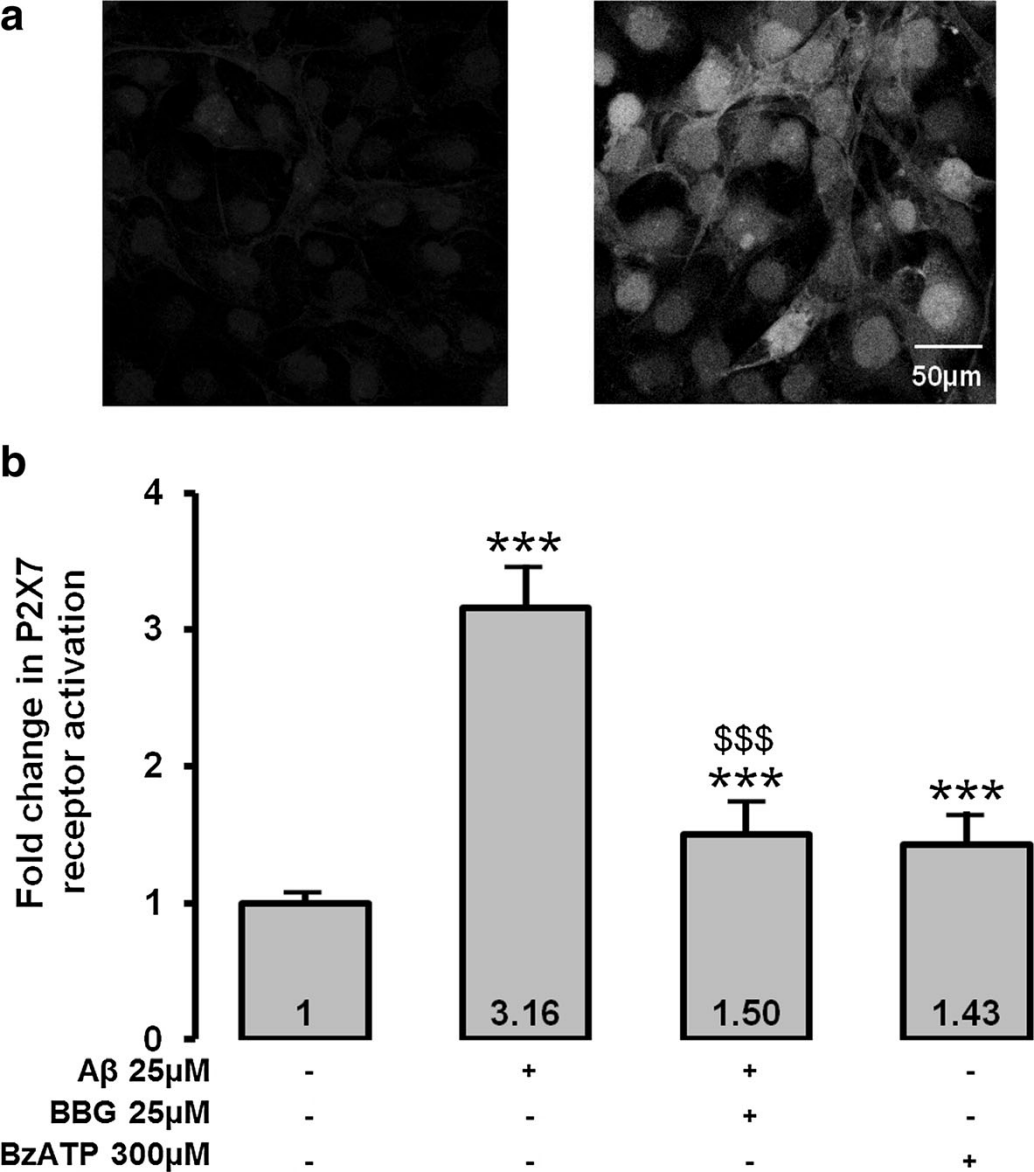
Expression and activation of P2X7 receptor in MIO-M1 cells. **a** Isotype control (*left*) and P2X7 receptor labeling (*right*). Cells were observed using confocal microscopy (200×). Pictures show mergence between nuclei and P2X7 staining. **b** P2X7 receptor activation using the YO-PRO-1 assay was evaluated after incubation of 25 μM Aβ for 48 h. BBG at 25 μM was used as a P2X7 receptor potent inhibitor and BzATP at 300 μM was used as a positive control. ***p < 0.001 compared to negative control; $$$*p* < 0.001 compared to Aβ

Figure 2 shows a strong labeling of MIO-M1 cell membranes using a specific anti-P2X7 antibody (Fig. 2a, right) compared to the isotype control antibody (Fig. 2a, left).

**Fig. 3:**
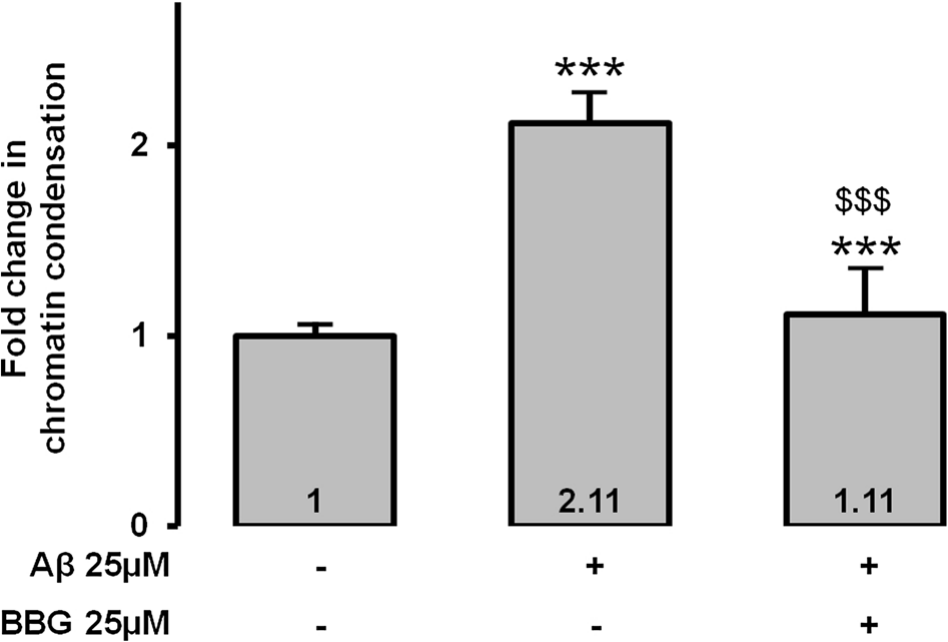
Chromatin condensation after incubation of 25 μM Aβ for 48 h. Chromatin condensation using Hoechst 33342 assay was evaluated after Aβ incubation for 48 h in MIO-M1 retinal Müller cells. BBG at 25 μM was used as a P2X7 receptor potent inhibitor. ***p < 0.001 compared to negative control; $$$*p* < 0.001 compared to Aβ

**Fig. 4:**
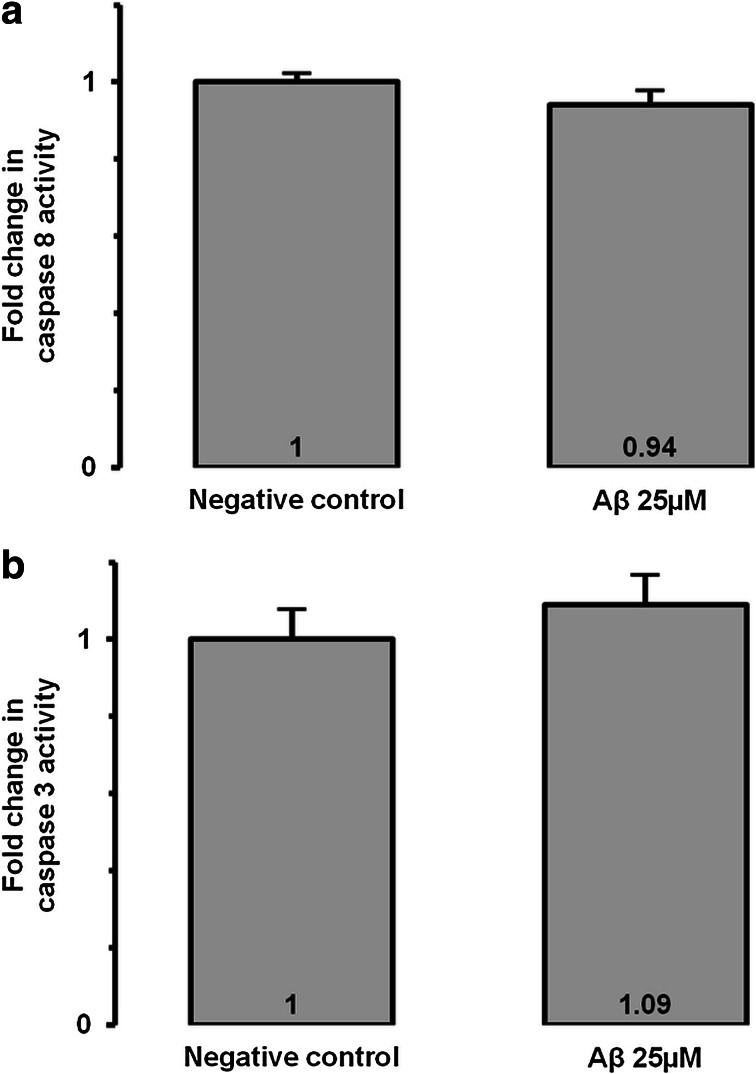
Caspase activation after incubation of 25 μM Aβ for 48 h. Caspase 8 (**a)** and caspase 3 (**b**) activation were evaluated after Aβ incubation for 48 h in MIO-M1 retinal Müller cells

Then, we analyzed P2X7 pore formation by the YO-PRO-1 assay. Fluorescence signal significantly increased (×3.16, *p* < 0.001) when MIO-M1 were incubated with Aβ compared to negative control (Fig. 2b). When the Aβ-incubated MIO-M1 were preincubated with the P2X7 inhibitor, BBG, the fluorescence signal significantly decreased (×0.48, *p* < 0.001) compared to Aβ-incubated MIO-M1 without preincubation, confirming activation of P2X7 by Aβ. We also observed that a specific P2X7 receptor activator, BzATP, increased P2X7 pore formation, which confirms the specificity of the YO-PRO-1 assay to evaluate P2X7 activation. We also studied chromatin condensation, an irreversible early phase of apoptosis assessed by the Hoechst 33342 assay, because even no loss of cell viability at 48 h does not mean no apoptosis at 48 h. In Fig. 3, Hoechst 33342 fluorescence was significantly increased (×2.11, *p* < 0.001) in Aβ-treated MIO-M1 compared to negative control. Preincubation of Aβ-treated MIO-M1 with BBG totally inhibited chromatin condensation compared to Aβ-treated MIO-M1 without preincubation increased (×0.53, *p* < 0.001), indicating the central role of P2X7 in the mediation of Aβ-induced condensation of Müller cell chromatin. Finally, we evaluated caspase activation. We focused our attention on caspase 8 and caspase 3. No significant difference was observed between Aβ-treated MIO-M1 compared to negative control (Fig. 4a, b).

Altogether, these data indicated that Aβ first alters the chromatin state of Müller cells without inducing cell death at 48 h.

### Protective Effects of EPA/DHA Associated in Fish Oil and BBG on Aβ-Cytotoxicity

**Fig. 5:**
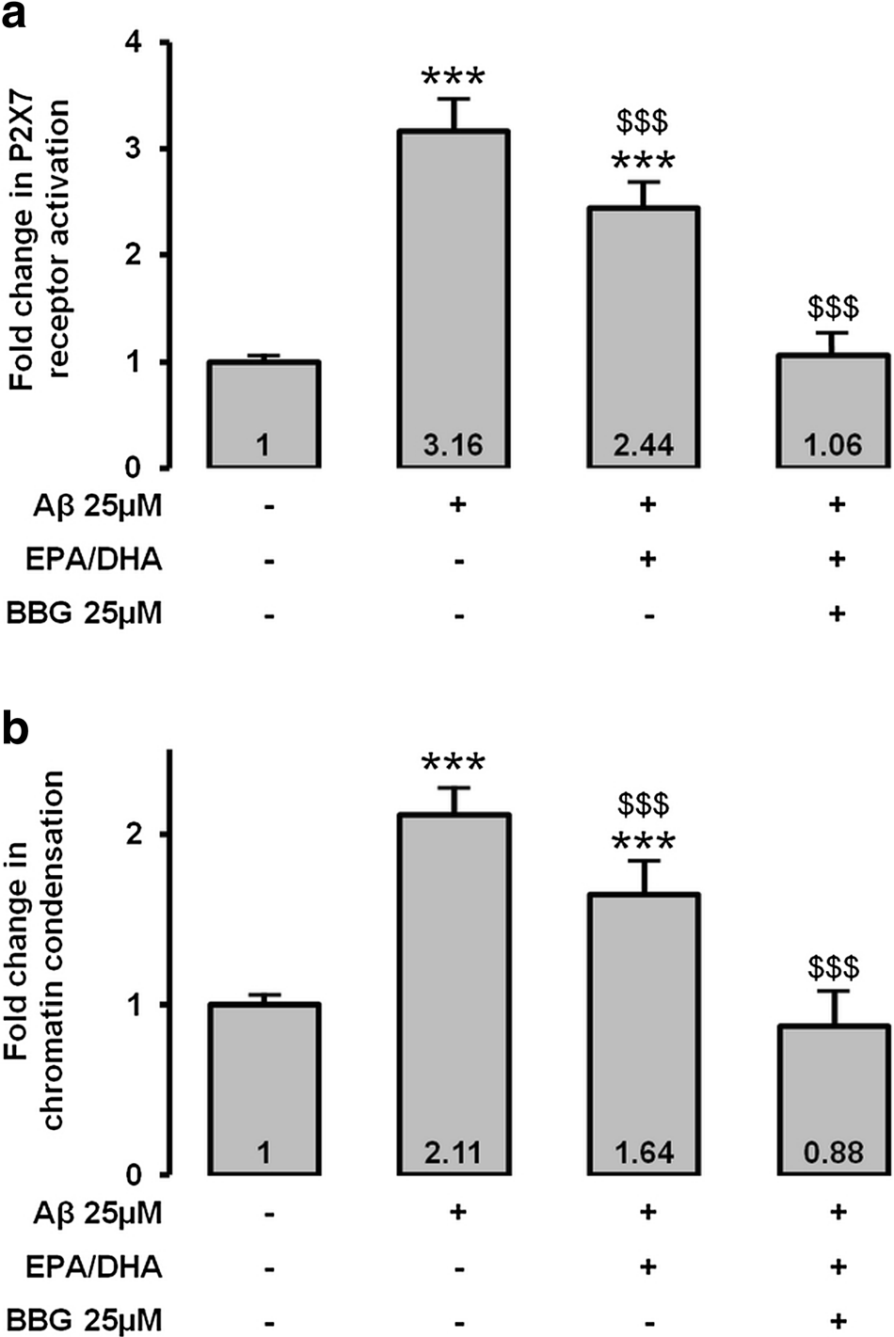
Aβ-induced apoptosis modulation with marine EPA and DHA lipid formulation. P2X7 receptor activation (**a**) and chromatin condensation (**b**) were evaluated after preincubation with fish EPA–DHA oil for 15 min and incubation of 25 μM Aβ for 48 h in MIO-M1 retinal Müller cells. BBG at 25 μM was used as a P2X7 receptor potent inhibitor. ***p < 0.001 compared to negative control; $$$*p* < 0.001 compared to Aβ

EPA and DHA have antioxidant and antiapoptotic roles in the retina, but their protective effects on Müller cells against Aβ remains undetermined. P2X7 pore formation was significantly decreased (×0.77, *p* < 0.001) when Aβ-treated MIO-M1 were preincubated with EPA–DHA fish oil compared to Aβ alone, but the signal remains significantly higher than in the negative control (Fig. 5a), suggesting that P2X7 receptor is a target for protective effects of EPA and DHA in Aβ-treated Müller cells. Preincubation of MIO-M1 with both EPA–DHA fish oil and BBG totally inhibited P2X7 pore formation (×0.33, *p* < 0.001), meaning that BBG and EPA–DHA fish oil act synergically.

Analysis of chromatin condensation, an irreversible early phase of apoptosis assessed by the Hoechst 33342 assay, showed a significant decrease (×0.78, *p* < 0.001) in Hoechst 33342 signal when the Aβ-treated MIO-M1 were preincubated with EPA–DHA fish oil compared to Aβ-treated MIO-M1 without preincubation (Fig. 5b). Preincubation of Aβ-treated MIO-M1 with both EPA–DHA fish oil and BBG totally inhibited chromatin condensation compared to Aβ-treated MIO-M1 with or without preincubation with EPA–DHA fish oil (×0.42, *p* < 0.001), confirming the P2X7 receptor-mediated deleterious effects of Aβ and the potential protective role of EPA–DHA against these effects in Müller cells.

## Discussion

Müller cells have been shown to be implicated in AMD [47, 48], and selective ablation of these cells led to photoreceptor apoptosis, blood–retinal barrier breakdown, and retinal neovascularization [49]. We studied the Aβ effects on MIO-M1 cells to study the impact of this peptide involved in AMD pathogenesis on Müller cells. The lack of necrosis in the cells was determined by no increase in LDH levels and low levels of propidium iodide (data not shown). Apoptosis was revealed by higher levels of YO-PRO-1 and Hoechst 33342 fluorescence signals in Aβ-incubated cells than in control cells. Therefore, we report that oligomerized Aβ induces apoptosis rather than necrosis on human retinal Müller cells (MIO-M1). P2X7 receptor is involved in oxidative stress, cell death, and inflammatory processes, all of which have been linked to AMD. Aβ-induced apoptosis appears to be P2X7 cell death receptor-dependent and caspase-independent, but further investigations are needed to confirm that. We showed for the first time that P2X7 receptor activation plays a pivotal role in Aβ-induced apoptosis in Müller cells. Indeed, P2X7 receptor inhibition using a specific antagonist (BBG) drastically decreased Aβ-induced apoptosis. Our results are in accordance with previous results that showed that P2X7 receptor blockade prevents photoreceptor cell apoptosis and confers neuroprotection in the brain of a rat model of Alzheimer’s disease [50, 51]. However, the mechanism by which BBG acts remains to be deeper studied. In our model, P2X7 activation was not associated with extrinsic caspase 8 activation, as previously described [52, 53]. Caspase 3, which is involved both in the extrinsic and the intrinsic pathways, was not activated in our model, meaning that Aβ peptide induces P2X7 activation leading to caspase-independent chromatin condensation in Müller cells. Apoptosis-inducing factor (AIF) translocates from mitochondria to nuclei in a caspase-independent fashion, leading to DNA fragmentation and chromatin condensation in cells undergoing apoptosis [54, 55]. It was previously described that Aβ-induced cell death was associated with AIF translocation [56, 57].

The Age-Related Eye Disease Study 2 (AREDS2) was a multi-center, randomized trial designed to assess the effects of oral supplementation of DHA and EPA on the progression to advanced AMD. The results of this trial showed that addition of DHA and EPA as ethyl esters did not further reduce the risk of progression to advanced AMD. In our model, EPA and DHA as triglycerides in fish oil had preventive effects towards P2X7 cell death receptor-dependent apoptosis induced by Aβ. We previously demonstrated that individual synthetic DHA or EPA are not as efficient as DHA and EPA associated in fish oils in the prevention of some ocular stresses [31]. Moreover, appropriate proportions of DHA/EPA seem to be needed to observe the most potent effect. Fatty acids contained in the lipid formulation we selected incorporate into retinal cell membranes [31], which can increase membrane fluidity and disrupt lipid rafts [58–61]. The activity of the numerous receptors expressed in lipid rafts, such as P2X7 receptor [62], may be modified. Effectively, we observed that Aβ-induced P2X7 receptor activation was reduced by our lipid formulation. This P2X7 cell death receptor blockade could occur through lipid raft disruption, as we previously showed that the EPA–DHA fish oil we used is able to modulate lipid rafts organization [63].

DHA (22:6 ω-3) is the precursor of EPA (20:5 ω-3), which is the omega-3 homologue of arachidonic acid (20:4 ω-6). Arachidonic acid is at the origin of pro-inflammatory mediators (prostaglandin E2); on the contrary, EPA is at the origin of anti-inflammatory mediators (prostaglandin E3) after metabolism by COX enzymes [64]. As we observed in a previous study, an increase in EPA can lead to a decrease in arachidonic acid in cell membranes and then to a decrease in the pro-inflammatory response [32]. It is thus suggested that the high content of EPA in our fish oil formulation could help in diminishing the inflammation associated to AMD. Fish EPA–DHA and BBG exerted synergic effects in the prevention of Aβ damages in our model. As the potential application of BBG as a neuroprotective therapy has already been suggested [50], the mixture of fish EPA–DHA and BBG opens further new strategic therapeutics.

## Conclusion

For the first time, our study showed that Aβ seems to induce caspase-independent apoptosis through P2X7 receptor activation in human retinal cells. We showed that marine lipid formulation containing EPA and DHA as triglycerides, in combination with BBG, a specific P2X7 receptor inhibitor, fully prevented Aβ cytotoxic effects in our model. Therefore, marine oils rich in EPA and DHA, in combination with a potent P2X7 receptor antagonist, could represent a promising efficient modulator of Aβ toxicity.
